# Parliament and Publications

**Published:** 1910-08-27

**Authors:** 


### Parliament and Publications.

### DEATH-RATE FROM TUBERCULOUS DISEASES.

A return recently furnished by the President of the Local Government Board states that the death rates from consumption and from other tuberculous diseases per 10,000 of the estimated population in each of the years 1901 to 1908 were as follows:—

| Years | Consumption | Other Tuberculous Disease |
|---|---|---|
| 1901 ... ... ... | 12.64 | 5.43 |
| 1902 ... ... ... | 12.33 | 5.08 |
| 1903 ... ... ... | 12.03 | 5.39 |
| 1904 ... ... ... | 12.36 | 5.41 |
| 1905 ... ... ... | 11.40 | 4.92 |
| 1906 ... ... ... | 11.50 | 4.94 |
| 1907 ... ... ... | 11.40 | 4.65 |
| 1908 ... ... ... | 11.15 | 4.68 |

The figures for the year 1909 are not yet tabulated.

### INTERNATIONAL CONGRESS OF PHYSIOTHERAPY.

In his report on the International Congress of Physiotherapy, held at Paris, the delegate of his Majesty's Government (H. Lewis Jones, M.D., F.R.C.P.) submits that a Congress in London, say in 1914 or 1915, to succeed that already arranged to be held in Berlin in 1912 would be likely to prove a success, and would also be valuable in the interests of British medicine.

### FAILURES OF MEDICAL PRACTITIONERS.

According to the annual report of the Inspector-General in Bankruptcy the failures of "Doctors of Medicine, Surgeons, etc.," under Bankruptcy and Deeds of Arrangement Acts last year was 41, the total liabilities being £74,216. In 1908 the number of failures was 25, with liabilities of £31,712; in 1907, 30, with liabilities £52,287; in 1906, 31, with liabilities £34,672; in 1905, 35, with liabilities £81,781.

### MORTALITY IN BACK-TO-BACK HOUSES.

A report on the relative mortality in through and back-to-back houses in certain towns in the West Riding of Yorkshire has been issued as a Government publication. The investigation was conducted by Dr. Darra Mair, and the statistical results obtained show that even relatively good types of back-to-back houses, when compared with through houses, have a death-rate from all causes taken together which is 15 to 20 per cent. in excess of the death-rate in through houses; although this excess is not evident in back-to-back houses built in blocks of four, possessing some degree of cross ventilation, unlike those built in continuous rows. It is noteworthy, however, that in back-to-back houses there is excessive mortality from certain important groups of diseases, whether these houses are built in blocks of four or in continuous rows. The groups of diseases thus showing excess are diseases of the chest, like bronchitis and pneumonia, and diseases especially associated with defective growth and development of the young child. Dr. Darra Mair's statistics show that it is the excess of mortality from these groups of diseases which is mainly responsible for the total excessive mortality in back-to-back houses. The statistics also show that the excessive mortality associated with back-to-back houses falls chiefly in childhood and old age. "Report on Back-to-Back Houses," by Dr. L. W. Darra Mair (Cd. 5314). Price 3d.

### THE WORK OF THE GOVERNMENT LABORATORY.

The report of the principal chemist of the Government laboratory upon the work of the laboratory for the year ended March 31, 1910, refers to several matters of interest to medical practitioners. A large increase is shown in the number of samples of tea submitted during the year under review as compared with the previous year. Of the 7,369 samples examined, 648, representing 1,673 packages, were rejected on account of the presence of foreign substances, or an excessive proportion of mineral matter, or as being unsound. The greater part of these, however (1,061 packages), consisted of "tea fluff," or dust, and was intended for use in the manufacture of caffeine, and not for consumption as "tea." Of the remaining packages, presented for home consumption but condemned, only 59 (representing approximately 500 lb.) were rejected as being "unfit for human food." The result of the Customs examination has been practically to stamp out the adulteration of tea prior to importation into the United Kingdom. Of 380 samples of "herb beers" examined 31 contained 3 per cent. but less than 4 per cent. of proof spirit; 6 contained 4 per cent. but less than 6 per cent. of proof spirit; 1 containing 6.2 per cent. was described as "ginger beer." Of 412 samples of beer and brewers' materials tested for the presence of arsenic 35 were found to contain it in excess of the limits laid down by the Royal Commission on Arsenical Poisoning—namely, the equivalent of one-hundredth of a grain of arsenious oxide per pound in the case of solids, or per gallon in the case of liquids. It is mentioned in the report that the effect of the "White Phosphorus Matches Prohibition Act, 1908," already has been practically to stamp out the use of white phosphorus so far as importations into the United Kingdom are concerned. Out of 647 samples of matches examined in the laboratory since the Act came into operation there were only seven instances in which the presence of white phosphorus was detected. The total number of analyses made in the Government laboratory during the year, including the samples examined at the testing and chemical stations, was 323,464.

